# Positive association between insulin resistance and fatty liver disease in psoriasis: evidence from a cross-sectional study

**DOI:** 10.3389/fimmu.2024.1388967

**Published:** 2024-04-23

**Authors:** Xiaoyuan Zhong, Dawei Huang, Rongfen Chen, Lingling Yao, Rui Ma, Yingyuan Yu, Yuxiong Jiang, Luyang Kong, Jiajing Lu, Ying Li, Yuling Shi

**Affiliations:** ^1^ Department of Dermatology, Shanghai Skin Disease Hospital, Tongji University School of Medicine, Shanghai, China; ^2^ Institute of Psoriasis, Tongji University School of Medicine, Shanghai, China

**Keywords:** psoriasis, fatty liver disease, insulin resistance, TyG, TyG-BMI

## Abstract

**Background:**

Fatty liver disease (FLD) is a common comorbidity of psoriasis and is often referred to as non-alcoholic fatty liver disease (NAFLD). However, the role of inflammation or insulin resistance (IR) in FLD is inconclusive. The study aims to explore whether FLD in psoriasis patients is more related to insulin resistance or systemic inflammation level.

**Methods:**

Data for this study were collected from the Shanghai Psoriasis Effectiveness Evaluation Cohort, a prospective cohort that examines psoriasis characteristics in the Chinese population. IR was assessed using the triglyceride glucose (TyG) and TyG-body mass index (TyG-BMI) indicators. Systemic non-specific inflammation was assessed using the neutrophil-to-lymphocyte ratio (NLR), derived neutrophil-to-lymphocyte ratio (dNLR), and systemic immune inflammation index (SII).

**Results:**

The analysis included a total of 647 patients. Subsequent logistic regression analysis revealed that NLR, dNLR, and SII were not significantly associated with FLD in psoriasis patients, while TyG and TyG-BMI showed significant associations with FLD. Subgroup analysis indicated that in the majority of subgroups, TyG and TyG-BMI were significantly associated with FLD, particularly TyG-BMI. Excluding individuals with methotrexate and acitretin resulted in consistent findings with the main analysis. Further analysis revealed a significantly higher diagnosis rate of metabolic-associated fatty liver disease (MAFLD) compared to NAFLD.

**Conclusions:**

Metabolic factors play a crucial role in FLD in patients with psoriasis, and TyG and TyG-BMI are potential predictors of FLD. Therefore, MAFLD can be recommend as a term to describe FLD in psoriasis patients.

**Trial registration:**

https://www.chictr.org.cn/showproj.html?proj=58256, identifier ChiCTR2000036186. A multi-center clinical study of systemic treatment strategies for psoriasis in Chinese population. Registered 31 August 2020.

## Introduction

Psoriasis is a chronic systemic inflammatory skin disease that extends beyond the skin and affects the function of many organs throughout the body. Fatty liver disease (FLD) is a common pathophysiological change in patients with psoriasis, and often describes as non-alcoholic fatty liver disease (NAFLD) which is a common comorbid disease of psoriasis and has been a concern for dermatologists (1, 2).In fact, NAFLD is an exclusive disease, there may be many more patients with FLD in patients with psoriasis than there is available evidences. In 2020, an international panel of experts proposed the metabolic-associated fatty liver disease (MAFLD) definition (3). MAFLD is a positive diagnosis that can be compared with other types of FLD, and may be an alternative to NAFLD (3, 4).

The mechanism of FLD in patients with psoriasis is not yet clear, but it is believed that higher systemic inflammation in patients with psoriasis affects insulin resistance (IR), thus aggravating hepatic steatosis (5, 6).Current research is limited to a single inflammatory factor or a single pro-inflammatory factor, and no studies have evaluated the effect of systemic inflammation on FLD in patients with psoriasis.

Indicators of neutrophil-to-lymphocyte ratio (NLR), derived neutrophil-to-lymphocyte ratio (dNLR), and systemic immune inflammation index (SII), which have been shown to correlate with systemic inflammation levels in a variety of diseases, are associated with FLD (6–9). Triglyceride-glucose (TyG) and triglyceride glucose-body mass index (TyG-BMI) are new indicators for evaluating IR, which have been verified in a number of studies to be consistent with or even better than traditional evaluation indicators (10–12). Therefore, the purpose of this study was to explore the association of inflammation or IR in patients with FLD using NLR, dNLR, SII, TyG, and TyG-BMI. Compare the differences between groups based on the diagnostic criteria for NAFLD and MAFLD, and further discuss suitable descriptive terms for psoriatic FLD.

## Materials and methods

### Study design

The study focused on analyzing patients with psoriasis who were enrolled in the Shanghai Psoriasis Effectiveness Evaluation Cohort (SPEECH) (13). SPEECH is an ongoing observational registry that involves multiple medical centers and aims to explore the unique characteristics of psoriasis in the Chinese population. Additionally, the registry seeks to identify appropriate diagnostic and treatment approaches for this specific demographic. The Ethics Committee of the Shanghai Skin Disease Hospital (#2020-36) thoroughly reviewed this study. All participants provided informed consent, and their privacy and confidentiality were carefully safeguarded throughout the study.

### Participants

All study participants were registried in the SPEECH cohort. Inclusion criteria were as follows: 1. Adults who had been diagnosed with moderate-to-severe plaque psoriasis; 2. FLD was assessed using ultrasound, and if the ultrasound was of poor quality or negative, the patient needed to undergo an additional abdominal computerized tomography; 3. Routine blood tests, fasting glucose, and triglyceride tests required venous blood samples to be collected in the early morning. Main exclusion criteria included lack of baseline information, assessment of FLD, or venous blood samples.

### Assessment of insulin resistance

The most accurate way to assess IR is the hyperinsulinemic-euglycemic clamp (HIEC) (14), but this method is very complex and difficult to apply in clinical practice outside of endocrinology. In addition, the Homeostasis Model Assessment-IR (HOMA-IR) is widely recognized, but the calculation relies on fasting insulin levels, which are not usually measured in routine diagnosis and treatment of psoriasis. This has led to the use and limitation of HOMA-IR in the psoriasis patient population. Recently, new indices known as TyG and TyG-BMI have been recognized as reliable and direct markers of IR (12, 15). TyG is calculated as follows: Ln [triglyceride (mg/dL) × fasting blood glucose (mg/dL)/2] (16); and TyG-BMI is calculated as TyG x BMI (kg/m2) (12). Therefore, TyG and TyG-BMI were used to assess the levels of IR in this study.

### Assessment of non-specific systemic inflammation

Non-specific systemic inflammation was assessed using blood-derived markers, which were measured alongside liver assessments. These included the common NLR, dNLR, and SII. NLR was calculated by dividing the neutrophil count by the lymphocyte count. The dNLR is calculated as the neutrophil count divided by the difference between the white blood cell count and the neutrophil count. The formula for SII is the platelet count multiplied by NLR.

### Statistical analysis

The primary method of analysis in this study is cross-sectional analysis. The group was divided into two categories based on the presence or absence of diffuse liver steatosis on imaging. Continuous variables that exhibit a normal distribution are reported as mean ± standard deviation (SD) and evaluated using the student t-test. Non-normally distributed continuous variables were presented with the median (interquartile range [IQR]) and compared using the Mann-Whitney U test. Categorical variables are expressed as frequency (%) and evaluated using Chi-square tests or Fisher’s exact tests.

The patients were subsequently stratified into three average groups based on the NLR, dNLR, SII, TyG and TyG-BMI respectively due to the significant variability in the data. These groups were further categorized as Q1-Q3 according to ascending order. Covariates included age, gender, BMI, smoking history, drinking history, hypertension, diabetes, psoriatic arthritis, duration of psoriasis, family history and PASI score based on previous literature (17). Three models were developed to investigate the correlation: Model 1 did not include any covariates, Model 2 only adjusted for age, gender, smoking history, drinking history, and BMI, and Model 3 included all covariates for analysis. When using TyG-BMI as the target variable, BMI was not used as a covariate. Odds ratios (OR) and 95% confidence intervals (95% CI) for different groups of inflammatory and IR indictors were calculated using univariate logistic regression and multivariate logistic regression. The correlation between continuous variables and FLD was demonstrated using forest plots. The association of inflammation markers and IR indictors with FLD in model 3 was verified by restricted cubic spline diagram (RCS), with knots=4 selected. Subgroup analysis was then conducted to further explore potential interactions and influencing factors. Receiver operating characteristic (ROC) curves were then used to assess the ability of inflammatory markers and IR markers to identify FLD in patients with psoriasis.

Causal mediation analysis was conducted using the ‘mediation’ package in R, and the bootstrap method with 500 simulations was employed to estimate the indirect effects, considering the current mainstream mechanism that believes inflammation leads to psoriatic FLD through the mediating effect of IR in model 3. Probit regression in the “mediation” package was used to analyze the mediating effect as sensitivity analysis. As acitretin and methotrexate (MTX) are the most commonly systemic oral drugs for psoriasis in China and have a significant effect on the liver, another sensitivity analysis was conducted, calculating OR values for patients who have never used MTX or acitretin.

Finally, differences between patients with FLD were compared based on the diagnostic criteria of NAFLD and MAFLD. A p-value <0.05 was considered statistically significant. All statistical analyses were performed using R version 4.2.1.

## Results

### Patient characteristics

**Figure 1:**
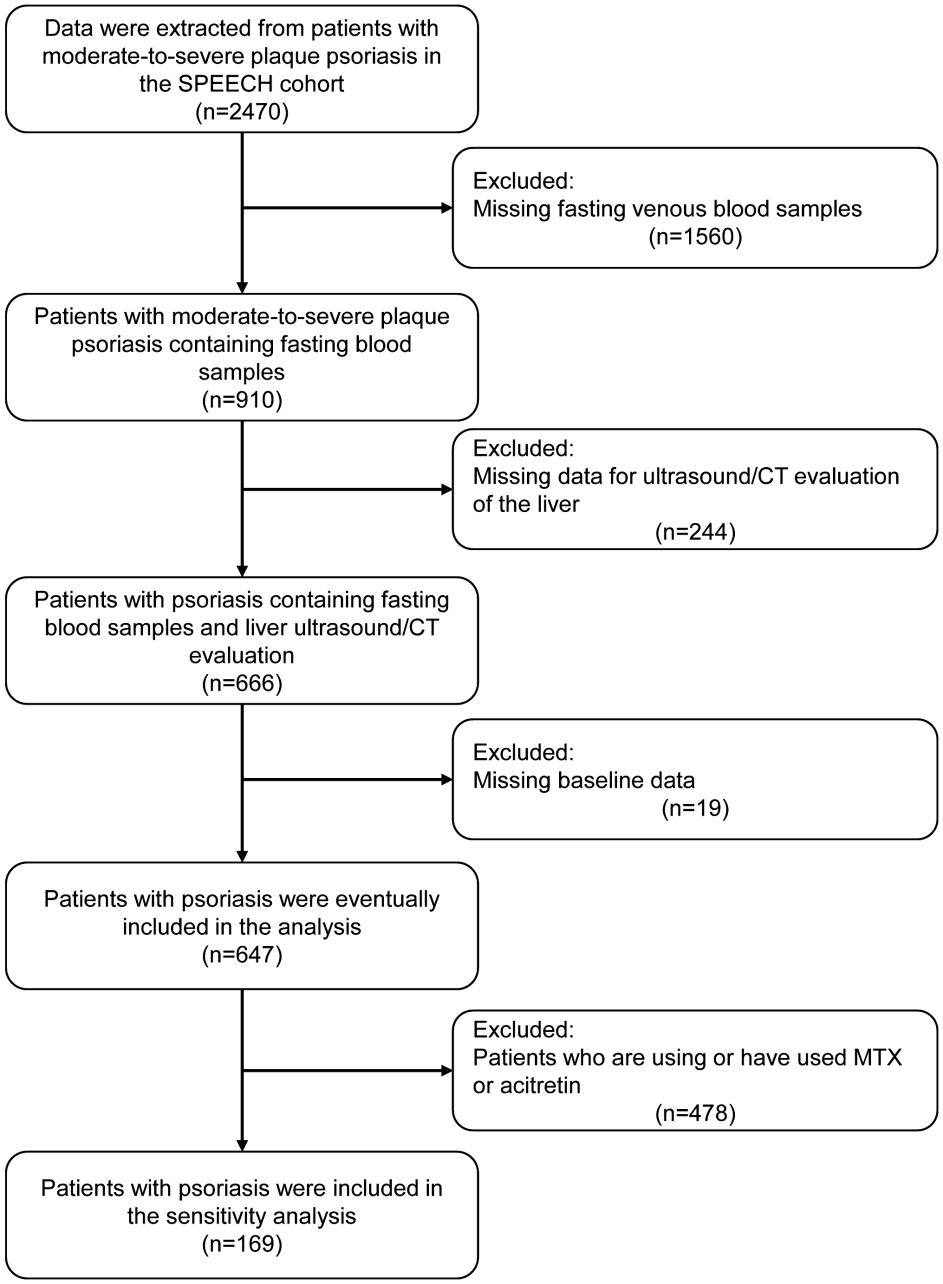
Flow chart of the study.

After screening the patient information of the SPEECH cohort according to the exclusion criteria, a total of 674 patients were included in the analysis (**Figure 1**). Among them, 308 patients (45.7%) were found to have liver steatosis in imaging examinations. Consistent with previous studies (18, 19), patients with FLD had a higher BMI, higher prevalence of hypertension, and higher levels of blood glucose, triglycerides, and IR. Surprisingly, patients with FLD were younger, and no significant differences in inflammatory markers were found between the two groups (**Table 1**).

**Table 1:** Baseline demographics and disease characteristics of psoriasis patients.

Parameter	FLD (n=308)	non-FLD (n=366)	*P* value
Demographics			
Gender, male, n (%)	251 (81.5%)	279 (76.2%)	0.097
Age, median (IQR)	55 (40, 65)	60 (45, 67)	0.008
Family history of psoriasis, n (%)	58 (18.8%)	60 (16.4%)	0.407
BMI (kg/m²), median (IQR)	26.1 (23.9, 29.0)	23.6 (21.2, 25.7)	< 0.001
Smoking, n (%)	140 (45.5%)	139 (38%)	0.050
Drinking, n (%)	67 (21.8%)	84 (23%)	0.710
Comorbidity			
Hypertension, n (%)	123 (39.9%)	109 (29.8%)	0.006
Type 2 diabetes mellitus, n (%)	56 (18.2%)	56 (15.3%)	0.317
PSA, n (%)	68 (22.1%)	75 (20.5%)	0.616
Evaluation of psoriasis			
Duration (years), median (IQR)	10 (6, 20)	10 (6, 20)	0.973
Prior-MTX, n (%)	135 (43.8%)	160 (43.7%)	0.976
Prior-acitretin, n (%)	160 (51.9%)	198 (54.1%)	0.577
BSA (%), median (IQR)	30 (15, 41)	25 (14, 40)	0.167
PASI, median (IQR)	15.3 (11.6, 21.0)	15.0 (11.5, 21.0)	0.318
Hematological results			
Leukocyte (*10^9^/L), median (IQR)	6.6 (5.6, 7.8)	6.2 (5.3, 7.7)	0.004
Thrombocyte (*10^9^/L), median (IQR)	211.5 (174.8, 263.3)	208 (170.3, 256.5)	0.343
Lymphocyte (*10^9^/L), median (IQR)	1.9 (1.5, 2.3)	1.8 (1.4, 2.1)	< 0.001
Neutrophil (*10^9^/L), median (IQR)	3.9 (3.3, 5.0)	3.9 (3.0, 4.9)	0.066
NLR, median (IQR)	2.1 (1.6, 2.9)	2.2 (1.6, 3.1)	0.320
dNLR, median (IQR)	1.5 (1.2, 2.0)	1.6 (1.2, 2.1)	0.571
SII, median (IQR)	449.0 (317.1, 668.7)	461.8 (305.4, 663.5)	0.738
Fasting blood glucose (mmol/L), median (IQR)	5.4 (4.9, 6.4)	5.1 (4.7, 5.7)	< 0.001
Triglyceride (mmol/L), median (IQR)	1.7 (1.2, 2.3)	1.3 (1.0, 1.7)	< 0.001
TyG, median (IQR)	9.4 (9.0, 9.8)	9.1 (8.7, 9.5)	< 0.001
TyG-BMI, median (IQR)	249.8 (222.6, 276.0)	215.9 (191.6, 235.8)	< 0.001

FLD, fatty liver disease; IQR, interquartile range; BMI, body mass index; PSA, psoriatic arthritis; MTX, methotrexate; BSA, body surface area; PSAI, psoriasis area and severity index; NLR, neutrophil-to-lymphocyte ratio; dNLR, derived neutrophil-to-lymphocyte ratio; SII, Systemic Immune Inflammation Index; TyG, triglyceride-glucose; TyG-BMI, triglyceride glucose-body mass index.

* represents the unit of counting.

### Association between non-specific indictors of systemic inflammation and insulin resistance and FLD in psoriasis

Previous studies have documented a significant association between systemic nonspecific inflammatory indicators such as NLR and SII and FLD in the population. However, our findings indicate that there is no significant association between NLR, dNLR, and SII and the occurrence of FLD in patients with psoriasis. In contrast, we found that TyG and TyG-BMI were significantly associated with FLD in psoriasis, with a dose-dependent correlation observed in Q2 and Q3 compared to Q1 (**Table 2**). Compared with TyG, TyG-BMI was more significantly associated with FLD because of its larger OR (TyG-BMI: Q3 OR=7.946, 95% CI=5.138-12.480; TyG: Q3 OR=2.704, 95% CI=1.750-4.206). Additionally, the forest plot demonstrated that, after adjustments for covariates, systemic nonspecific inflammatory indicators exhibited a low association with FLD. In contrast, TyG and TyG-BMI showed a significant positive correlation with FLD in psoriasis patients in all models (**Supplementary Figure 1**). These results were further confirmed in the RCS (**Supplementary Figure 2**).

**Table 2:** Association between non-specific indictors of systemic inflammation and insulin resistance and fatty liver disease in psoriasis.

Exposures	Model 1^a^ Crude OR (95% CI)	Model 2^b^ Adjusted OR (95% CI)	Model 3^c^ Adjusted OR (95% CI)
NLR			
Q1	Ref.	Ref.	Ref.
Q2	1.123 (0.775-1.627)	1.055 (0.705-1.579)	1.038 (0.690-1.562)
Q3	0.881 (0.607-1.279)	0.932 (0.620-1.401)	0.897 (0.591-1.360)
dNLR			
Q1	Ref.	Ref.	Ref.
Q2	1.206 (0.832-1.750)	1.085 (0.726-1.622)	1.049 (0.697-1.576)
Q3	0.916 (0.631-1.329)	0.933 (0.623-1.399)	0.887 (0.586-1.343)
SII			
Q1	Ref.	Ref.	Ref.
Q2	0.843 (0.581-1.222)	0.780 (0.521-1.165)	0.767 (0.510-1.150)
Q3	0.898 (0.620-1.301)	0.967 (0.646-1.446)	0.932 (0.618-1.405)
TyG			
Q1	Ref.	Ref.	Ref.
Q2	1.756 (1.181-2.623)	1.817 (1.179-2.815)	1.871 (1.206-2.919)
Q3	3.131 (2.136-4.624)	2.621 (1.730-3.999)	2.704 (1.750-4.206)
TyG-BMI			
Q1	Ref.	Ref.	Ref.
Q2	2.495 (1.666-3.769)	2.575 (1.710-3.913)	2.584 (1.708-3.944)
Q3	8.189 (5.400-12.603)	8.300 (5.438-12.860)	7.946 (5.138-12.480)

OR, odds ratio; CI, confidence interval; NLR, neutrophil-to-lymphocyte ratio; dNLR, derived neutrophil-to-lymphocyte ratio; SII, systemic immune inflammation index; TyG, triglyceride-glucose; TyG-BMI, triglyceride glucose-body mass index; Ref, reference.

a No covariates analyzed.

b Covariates analyzed adjusted for age, gender, smoking, drinking, and BMI.

c All covariates analyzed.

**Figure 2:**
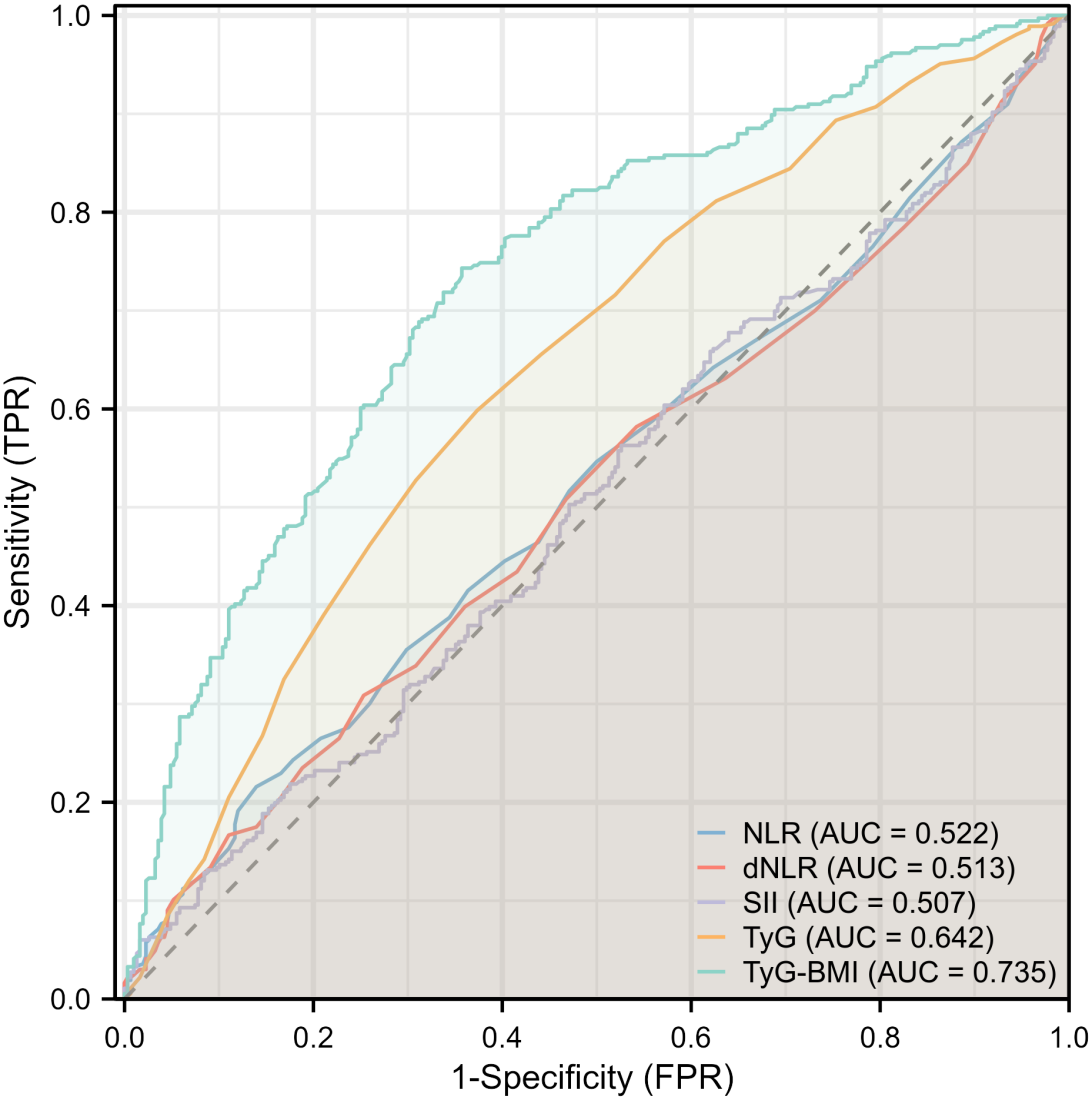
Forest plot depicting multivariate logistic regression results for inflammatory and insulin resistance indicators.

Given the significant association between TyG and TyG-BMI with FLD in patients with psoriasis, further subgroup analysis was conducted to determine the relationship (**Table 3**). The results of the subgroup analysis indicated that TyG and TyG-BMI remained significantly associated with FLD in the majority of subgroups (P for interaction > 0.05). It is worth noting that in patients with elevated triglycerides, the association between TyG and FLD was weakened, but TyG-BMI still maintained a good correlation. Subsequent ROC analysis revealed that the area under the curve (AUC) for TyG and TyG-BMI was larger than that for NLR, dNLR, and SII. The AUC of TyG-BMI was the largest at 0.735. These findings suggest that TyG-BMI is superior for determining FLD in patients with psoriasis (**Figure 2**).

**Table 3:** Subgroup analysis between indictors and fatty liver disease in psoriasis patients.

Subgroup	TyG	*P* value	*P* for interaction	TyG-BMI	*P* value	*P* for interaction
**Gender**			0.211			0.523
Male	2.077 (1.578-2.770)	<0.001		1.025 (1.015-1.037)	<0.001	
Female	2.373 (1.347-4.381)	0.004		1.022 (1.017-1.027)	<0.001	
**Age (years)**			0.012			0.176
<45	2.800 (1.817-4.496)	<0.001		1.025 (1.017-1.033)	<0.001	
≥45	1.930 (1.417-2.657)	<0.001		1.021 (1.015-1.027)	<0.001	
**Family history of psoriasis**			0.234			0.453
No	2.290 (1.677-3.079)	<0.001		1.022 (1.017-1.028)	<0.001	
Yes	1.790 (1.056-3.149)	0.035		1.025 (1.014-1.038)	<0.001	
**Smoking**			0.031			0.026
Never	2.348 (1.326-3.356)	<0.001		1.021 (1.015-1.027)	<0.001	
Past or current	1.979 (1.370-2.919)	<0.001		1.027 (1.019-1.036)	<0.001	
**Drinking**			0.735			0.943
Never	1.856 (1.406-2.479)	<0.001		1.021 (1.016-1.026)	<0.001	
Past or current	3.530 (2.063-6.395)	<0.001		1.030 (1.019-1.043)	<0.001	
**Type 2 diabetes mellitus**			0.492			0.794
No	2.444 (1.819-3.330)	<0.001		1.026 (1.020-1.031)	<0.001	
Yes	1.531 (0.899-2.682)	0.124		1.009 (0.999-1.020)	0.084	
**Hypertension**			0.050			0.184
No	2.397 (1.740-3.359)	<0.001		1.027 (1.021-1.033)	<0.001	
Yes	1.658 (1.113-2.521)	0.015		1.014 (1.006-1.022)	<0.001	
**BMI (kg/m²)**			<0.001			0.971
<24	2.100 (1.396-3.262)	0.001		1.026 (1.014-1.039)	<0.001	
≥24	1.804 (1.303-2.534)	0.001		1.020 (1.012-1.027)	<0.001	
**PSA**			0.347			0.892
No	2.213 (1.676-2.964)	<0.001		1.023 (1.017-1.028)	<0.001	
Yes	2.003 (1.150-3.613)	0.017		1.023 (1.013-1.035)	<0.001	
**Triglyceride**^a^			0.010			0.076
Normal	1.835 (1.204-2.832)	0.005		1.024 (1.018-1.031)	<0.001	
Borderline	0.954 (0.395-2.300)	0.916		1.011 (1.002-1.022)	0.026	
HTG	1.293 (0.699-2.576)	0.435		1.019 (1.007-1.024)	0.004	

BMI, body mass index; PSA, psoriatic arthritis; TyG, triglyceride-glucose; TyG-BMI, triglyceride glucose-body mass index; HTG, hypertriglyceridemia.

a Fasting plasma triglyceride concentrations were categorized as normal (<150 mg/dL), borderline (150-199 mg/dL), high triglyceride (HTG>200 mg/dL).

### Sensitivity analysis

First, we used causal mediation analysis and probit regression to examine the overall effects of NLR, dNLR, and SII on FLD in psoriasis patients. We then analyzed the indirect effects mediated by TyG and TyG-BMI, as well as the direct effects of inflammatory factors. The analysis revealed that the total effect of NLR on FLD in psoriasis was not significant for both TyG (coefficient -0.02125, p=0.108) and TyG-BMI (coefficient -0.02114, p=0.108). The indirect effects of NLR on FLD through TyG (coefficient -0.00154, P=0.404) and TyG-BMI (coefficient -0.00199, p=0.280) were also not significant. A similar pattern was observed for the direct effects. The proportion mediated was 7.3% (95% CI: -30.2, 94.1) for TyG and 9.4% (95% CI: -30.1, 96.5) for TyG-BMI, indicating a low mediation effect (**Supplementary Table 1**). Similar results were observed in dNLR and SII.

Given the widespread use of MTX and acitretin in China, even in the biologics era, these drugs continue to be the first-line treatment for patients with moderate-to-severe psoriasis in the country. Therefore, we excluded patients who had previously used MTX and acitretin, leaving only 169 patients in our cohort. In logistic regression analysis, similar to the main analysis, higher levels of TyG and TyG-BMI were found to be significantly associated with FLD in psoriasis, while NLR, dNLR, and SII showed no associations with FLD in psoriasis (**Supplementary Table 2**). This suggests that the results of the main analysis are robust.

### NAFLD and MAFLD

According to the diagnostic criteria for NAFLD and MAFLD (**Supplementary Figure 3**), 308 patients with hepatic steatosis detected on imaging were diagnosed. In our cohort, a total of 308 patients had radiographic evidence of hepatic steatosis (47.6%), with a diagnosis rate of 39.1% for NAFLD and 46.4% for MAFLD. Therefore, the prevalence of MAFLD was significantly higher than that of NAFLD (**Supplementary Table 3**). When comparing the characteristics of the two diagnostic populations, patients with MAFLD had a higher history of alcohol consumption and smoking than those with NAFLD. In addition, patients with MAFLD showed higher systemic non-specific inflammation and IR, although there was no statistical difference.

## Discussion

Comorbidities of psoriasis affecting multiple systems have received extensive clinical attention, including cardiovascular disease, obesity, diabetes, dyslipidemia, NAFLD, etc (20). FLD is characterized by the accumulation of lipids in the cytoplasm of hepatocytes. Currently, FLD is believed to be closely linked to IR and various inflammatory pathways. Systemic inflammation and metabolic disorders in psoriasis patients can exacerbate IR and worsen hepatic steatosis (21, 22). Our results are consistent with this, as patients with radiographic evidence of hepatic steatosis had higher levels of BMI, hypertension, fasting blood glucose, triglycerides, TyG and TyG-BMI compared to those without FLD.

In this cross-sectional study, we investigated the association of IR and non-specific systemic inflammatory conditions on FLD in patients with moderate-to-severe plaque psoriasis for the first time. Our results based on subgroup analysis and ROC indicate that systemic nonspecific indicators such as SII, NLR, and dNLR showed no significant association with FLD in patients with psoriasis. In contrast, IR-related indicators TyG and TyG-BMI were significantly associated with FLD in patients with psoriasis. Among these, TyG-BMI exhibited a higher AUC value and is expected to be a predictor of FLD in patients with psoriasis. Therefore, we suggest that metabolic factors play a more significant role in FLD in patients with moderate-to-severe plaque psoriasis than systemic inflammation.

IR is characterized by impaired downstream effects of insulin signaling in target tissues, primarily the liver, muscle, and adipose tissue (23). It is implicated in the progression of diseases such as FLD, as well as the advancement of liver fibrosis. Several studies have demonstrated an independent positive association between TyG-BMI and NAFLD (24–26). Our study indicates that TyG and TyG-BMI can be used as potential predictors of FLD in patients with psoriasis. There are some studies that have highlighted the significant value of indices such as BMI, TyG, and TyG-BMI, in identifying the risk of NAFLD and MAFLD. Some studies have proposed that TyG-WC, TyG-WHtR, and TyG-BMI could be used for early screening of NAFLD and MAFLD (24). Furthermore, these three parameters, along with HOMA-IR, were found to be more suitable for assessing metabolic risk and monitoring disease progression in NAFLD patients (24, 27). On comparison with HOMI-IR or the gold standard HIEC, TyG and TyG-BMI offer the advantages of convenience and accessibility, and can be considered as early screening tools for hepatic steatosis in patients with psoriasis.

Previous studies have demonstrated the effectiveness of SII, NLR, and dNLR in evaluating the severity of psoriasis disease activity and treatment effectiveness (28, 29). Some studies also have indicated their association with liver steatosis and NAFLD. Interestingly, our findings suggest that no significant association was found with NLR, dNLR and SII. This study utilized these three indicators to draw novel conclusions, which may be attributed to the fact that previous studies were conducted on a broader population and may not have specifically focused on patients with psoriasis. In addition, hepatic steatosis is closely linked to genetic and environmental factors. Studies have shown that dietary components and dietary habits can increase an individual’s susceptibility to NAFLD (30). Diets high in fat, salt, and sugar can contribute to hepatic steatosis, while dietary flavonoid intake acts as a protective factor (31). The dietary patterns of Chinese individuals differ somewhat from those of Europeans and Americans. Therefore, future studies should fully consider the heterogeneity of population and environmental factors.

It is estimated that the prevalence of NAFLD in the global population is 32.4% (32). Numerous studies have demonstrated heterogeneity in the pathogenesis of NAFLD (33). However, the emphasis of NAFLD on the role of alcohol, viral hepatitis, and other diseases in hepatic steatosis no longer accurately reflects the nature of the disease. In 2020, some scholars proposed the concept of MAFLD, which highlights the significant role of obesity, insulin resistance, dyslipidemia, diabetes, and systemic low inflammatory response in the occurrence and progression of fatty liver disease (4). In our study, the prevalence of NAFLD and MAFLD was 39.1% and 46.4%, respectively, significantly higher than that of the general population. The diagnosis rate of MAFLD was significantly higher than that of NAFLD in patients with psoriasis. As a result, FLD in patients with psoriasis may be significantly underestimated in the current understanding of NAFLD as the primary comorbidity in patients with psoriasis. Based on these results, considering the high incidence of viral hepatitis in China (34), NAFLD may not accurately reflect the specific situation of FLD in psoriasis patients, and MAFLD may be a viable alternative. However, the current evidence for MAFLD in the context of psoriasis is limited, and future high-quality studies with larger sample sizes are necessary to validate our findings.

Overall, our study assessed the association of metabolic and inflammatory factors in FLD in Chinese patients with psoriasis. Furthermore, we utilized the TyG and TyG-BMI to evaluate IR. These indicators are convenient, rapid, practical, and easily promotable in clinical practice. Therefore, our study may contribute to the clinical diagnosis and treatment of psoriasis with FLD.

This study has several limitations. Firstly, FLD was diagnosed solely through imaging and not histology. And the inflammatory indicators evaluated in the patients were non-specific, and did not include IL-17, IL-23, and other specific inflammatory indicators. Future studies should consider including specific indicators for research. Additionally, the patients in this study were predominantly middle-aged to elderly individuals with moderate-to-severe conditions, indicating a potential for selection bias. Furthermore, only TyG and TyG-BMI were used to assess IR, instead of the commonly used the gold standard HIEC. Finally, the cross-sectional nature of this study precludes the ability to make causal inferences. There is a lack of prospective studies on FLD in psoriasis, and future research should focus on larger sample sizes and higher-quality studies to validate our findings.

## Conclusion

Our study suggests that metabolic factors play a significant role in the development of FLD in patients with moderate-to-severe psoriasis. In understanding FLD in psoriasis, the term NAFLD may not fully encompass the condition of FLD in psoriasis in China. Therefore, to increase dermatologists’ awareness of psoriasis with FLD, we recommend including metabolic factors in the assessment of hepatic steatosis, and describing comorbidities in psoriasis in terms of MAFLD. We found that both TyG and TyG-BMI showed a significant association with FLD in patients with psoriasis and may serve as potential predictors. A more comprehensive approach to managing psoriasis is necessary in the future, incorporating metabolic assessments, interventions, and dietary modifications to manage or prevent hepatic steatosis in psoriasis.

## Data availability statement

The original contributions presented in the study are included in the article/**Supplementary Material**. Further inquiries can be directed to the corresponding authors.

## Ethics statement

The studies involving humans were approved by The Ethical Committee of Shanghai Skin Disease Hospital. The studies were conducted in accordance with the local legislation and institutional requirements. The participants provided their written informed consent to participate in this study.

## Author contributions

XZ: Writing – original draft. DH: Conceptualization, Investigation, Visualization, Writing – original draft. RC: Investigation, Writing – original draft. LY: Writing – original draft. RM: Visualization, Writing – original draft. YY: Writing – original draft. YJ: Conceptualization, Writing – original draft. LK: Writing – original draft. JL: Writing – review & editing. YL: Methodology, Resources, Writing – review & editing. YS: Writing – original draft, Writing – review & editing.

## Supplementary material

Supplementary Figure 1 Receiver operating characteristic analysis comparing hepatic steatosis indicators in psoriasis.

Supplementary Figure 2 Restricted cubic spline of non-specific inflammatory and insulin resistance indicators and fatty liver disease in psoriasis.

Supplementary Figure 3 Diagnostic criteria for non-alcoholic fatty liver disease and metabolic-associated fatty liver disease.
