# Supplementary figures and images for: Positive association between insulin resistance and fatty liver disease in psoriasis: evidence from a cross-sectional study
Source: Front Immunol. 2024 Apr 23;15:1388967. doi: 10.3389/fimmu.2024.1388967 (PMC11074461; doi:10.3389/fimmu.2024.1388967)

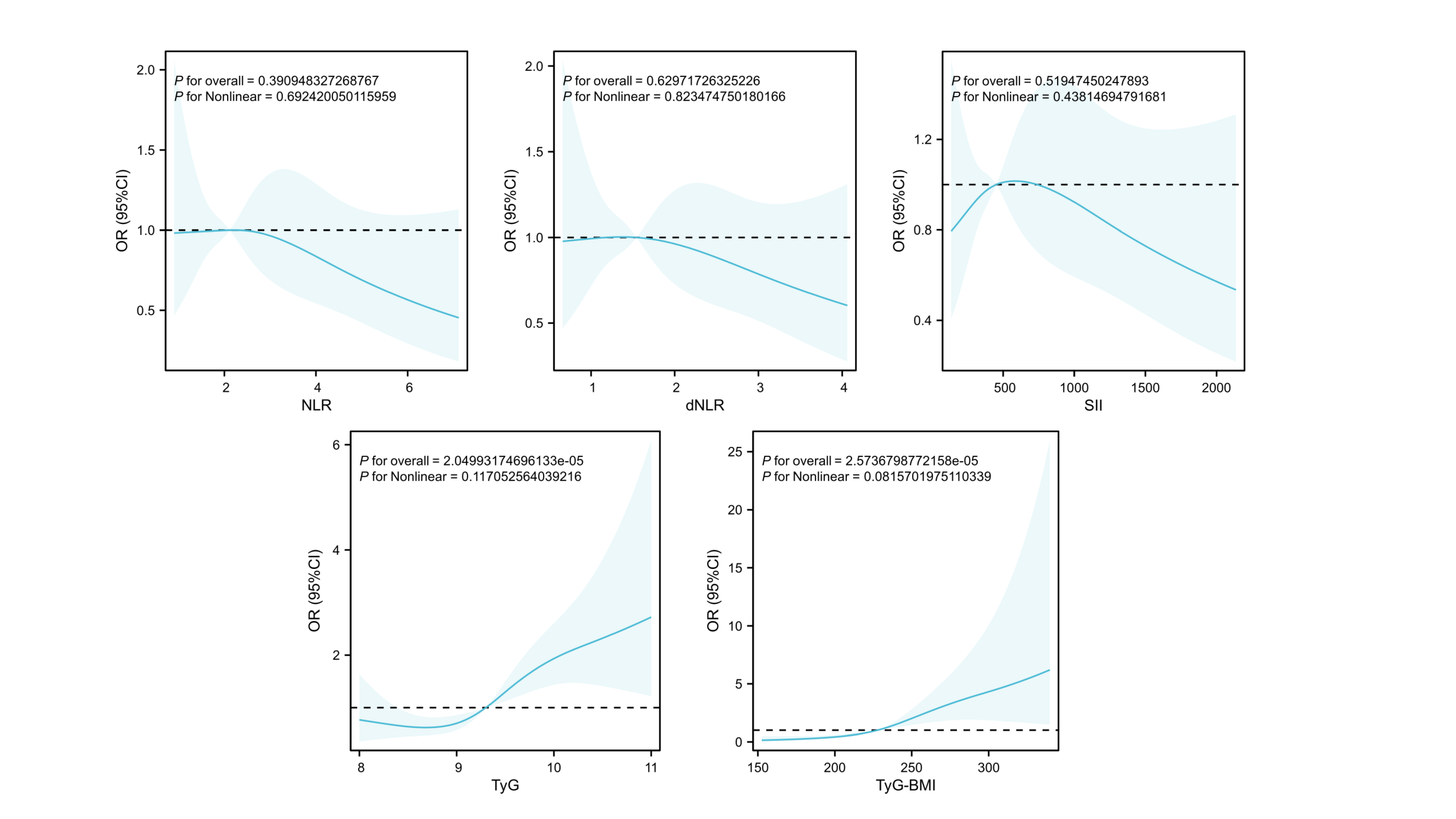

Supplement: Supplementary Figure 1 — Receiver operating characteristic analysis comparing hepatic steatosis indicators in psoriasis. [file Image_1.tif]

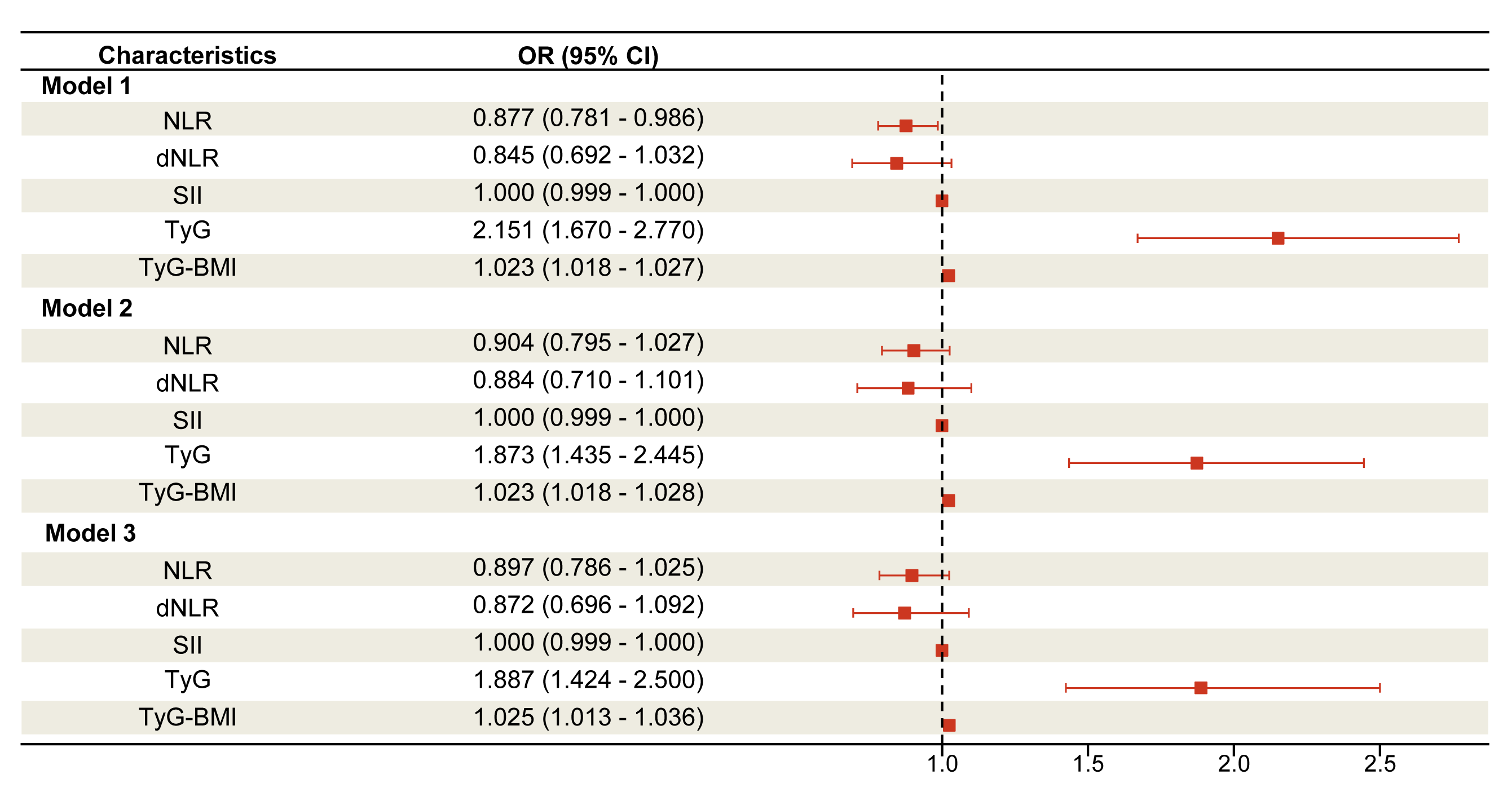

Supplement: Supplementary Figure 2 — Restricted cubic spline of non-specific inflammatory and insulin resistance indicators and fatty liver disease in psoriasis. [file Image_2.tif]

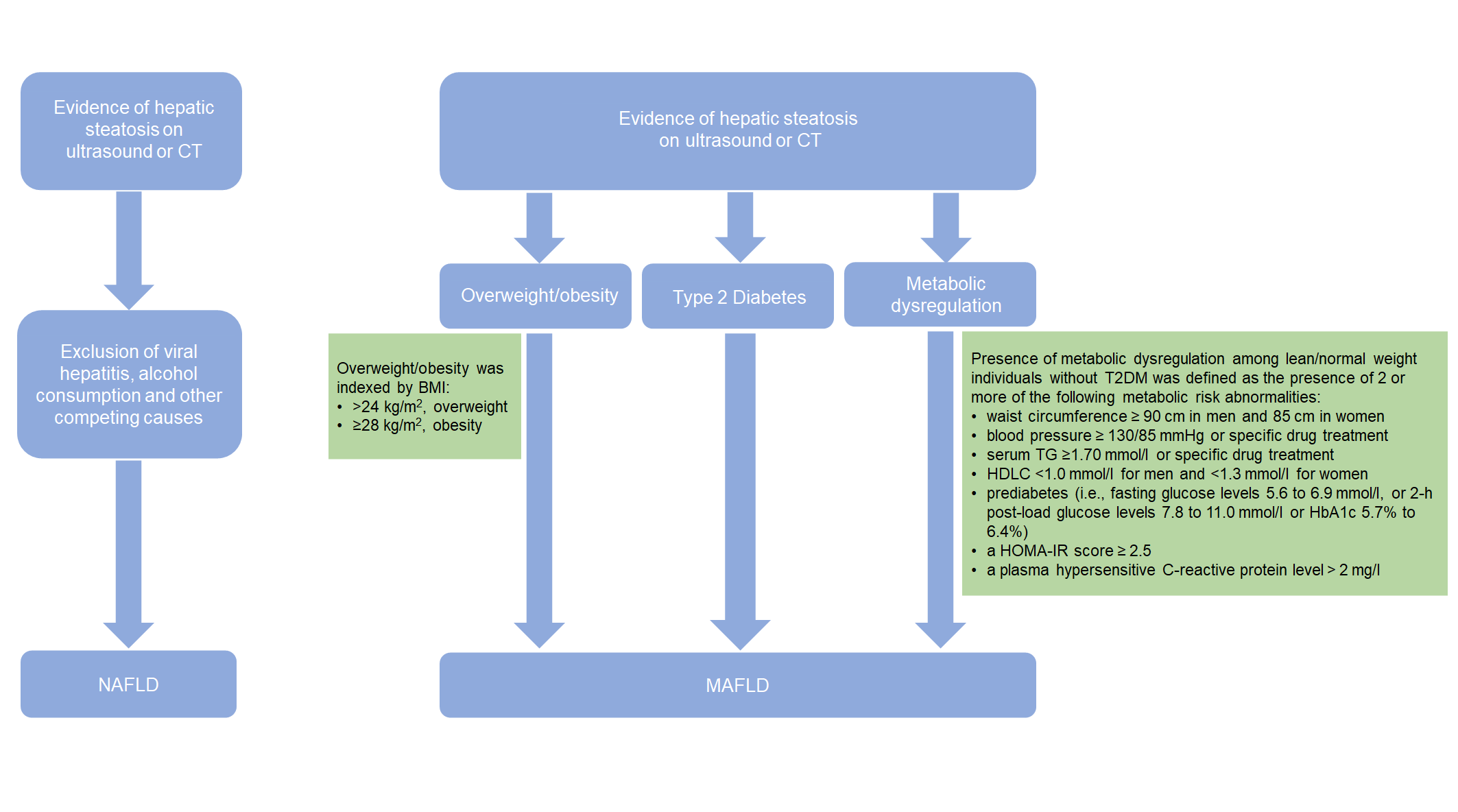

Supplement: Supplementary Figure 3 — Diagnostic criteria for non-alcoholic fatty liver disease and metabolic-associated fatty liver disease. [file Image_3.jpeg]
