# Supplementary material for: Positive association between insulin resistance and fatty liver disease in psoriasis: evidence from a cross-sectional study
Source: Front Immunol. 2024 Apr 23;15:1388967. doi: 10.3389/fimmu.2024.1388967 (PMC11074461; doi:10.3389/fimmu.2024.1388967)
Supplement: Supplementary file 4 [file Table_1.docx]

**Table S1. Effects of indictors on fatty liver disease in psoriasis patients: causal mediation analysis and probit regression outcomes.**

| Independent variable | Mediator | Total effect | | Indirect effect | | Direct effect | | Proportion mediated, % (95% CI) |
| --- | --- | --- | --- | --- | --- | --- | --- | --- |
|  |  | Coefficient (95% CI) | *P* value | Coefficient (95% CI) | *P* value | Coefficient (95% CI) | *P* value |  |
| **NLR** | TyG | -0.021 (-0.048, 0.003) | 0.108 | -0.002 (-0.006, 0.003) | 0.404 | -0.020 (-0.045, 0.005) | 0.116 | 7.3 (-30.2, 94.1) |
|  | TyG-BMI | -0.021 (-0.047, 0.004) | 0.108 | -0.002 (-0.007, 0.002) | 0.280 | -0.019 (-0.045, 0.006) | 0.124 | 9.4 (-30.1, 96.5) |
| **dNLR** | TyG | -0.027 (-0.071, 0.015) | 0.236 | -0.003 (-0.013, 0.006) | 0.472 | -0.024 (-0.067, 0.020) | 0.284 | 9.8 (-85.4, 197.3) |
|  | TyG-BMI | -0.026 (-0.071, 0.015) | 0.244 | -0.003 (-0.013, 0.004) | 0.388 | -0.023 (-0.066, 0.020) | 0.296 | 11.9 (-118.0, 169.9) |
| **SII** | TyG | -0.000 (-0.000, 0.000) | 0.372 | -0.000 (-0.000, 0.000) | 0.104 | -0.000 (-0.000, 0.000) | 0.508 | 22.9 (-144.9, 203.1) |
|  | TyG-BMI | -0.000 (-0.000, 0.000) | 0.372 | -0.000 (-0.000, -0.000) | 0.044 | -0.000 (-0.000, 0.000) | 0.540 | 29.6 (-191.2, 253.0) |

CI, confidence interval, NLR, neutrophil-to-lymphocyte ratio, dNLR, derived neutrophil to lymphocyte ratio, SII, Systemic Immune Inflammation Index.
