# Supplementary material for: Positive association between insulin resistance and fatty liver disease in psoriasis: evidence from a cross-sectional study
Source: Front Immunol. 2024 Apr 23;15:1388967. doi: 10.3389/fimmu.2024.1388967 (PMC11074461; doi:10.3389/fimmu.2024.1388967)
Supplement: Supplementary file 5 [file Table_2.docx]

**Table S2. Association between indicators and fatty liver disease in psoriasis: sensitivity analysis outcomes (n=169).**

| **Exposures** | **Model 1^a^**  **Crude OR (95% CI)** | **Model 2^b^**  **Adjusted OR (95% CI)** | **Model 3^c^**  **Adjusted OR (95% CI)** |
| --- | --- | --- | --- |
| **NLR** |  |  |  |
| Q1 | Ref. | Ref. | Ref. |
| Q2 | 1.241 (0.595-2.605) | 1.232 (0.544-2.813) | 1.199 (0.516-2.806) |
| Q3 | 0.639 (0.301-1.342) | 0.563 (0.243-1.280) | 0.532 (0.218-1.268) |
| **dNLR** |  |  |  |
| Q1 | Ref. | Ref. | Ref. |
| Q2 | 1.618 (0.766-3.458) | 1.875 (0.821-4.380) | 2.024 (0.853-4.932) |
| Q3 | 0.702 (0.333-1.467) | 0.686 (0.299-1.556) | 0.746 (0.312-1.772) |
| **SII** |  |  |  |
| Q1 | Ref. | Ref. | Ref. |
| Q2 | 0.930 (0.439-1.967) | 0.869 (0.381-1.968) | 0.944 (0.401-2.222) |
| Q3 | 0.875 (0.418-1.828) | 0.839 (0.373-1.883) | 0.857 (0.367-1.994) |
| **TyG** |  |  |  |
| Q1 | Ref. | Ref. | Ref. |
| Q2 | 1.212 (0.542-2.730) | 1.011 (0.419-2.440) | 1.225 (0.478-3.176) |
| Q3 | 3.909 (1.824-8.666) | 2.805 (1.221-6.604) | 4.082 (1.603-10.880) |
| **TyG-BMI** |  |  |  |
| Q1 | Ref. | Ref. | Ref. |
| Q2 | 1.816 (0.814-4.179) | 1.655 (0.727-3.874) | 1.931 (0.792-4.901) |
| Q3 | 11.000 (4.640-28.031) | 11.948 (4.901-31.612) | 15.023 (5.470-45.922) |

OR, odds ratio; CI, confidence interval; NLR, neutrophil-to-lymphocyte ratio; dNLR, derived neutrophil-to-lymphocyte ratio; SII, systemic immune inflammation index; TyG, triglyceride-glucose; TyG-BMI, triglyceride glucose-body mass index; Ref, reference.

^a^ No covariates analyzed.

^b^ Covariates analyzed adjusted for age, gender, smoking, drinking, and BMI.

^c^ All covariates analyzed.
