# Supplementary material for: Positive association between insulin resistance and fatty liver disease in psoriasis: evidence from a cross-sectional study
Source: Front Immunol. 2024 Apr 23;15:1388967. doi: 10.3389/fimmu.2024.1388967 (PMC11074461; doi:10.3389/fimmu.2024.1388967)
Supplement: Supplementary file 6 [file Table_3.docx]

**Table S3. Comparison of demographics and disease characteristics in patients with NAFLD and MAFLD psoriasis.**

| **Parameter** | **NAFLD (n=253)** | **MAFLD (n=300)** | ***P* value** |
| --- | --- | --- | --- |
| **Demographics** |  |  |  |
| Gender, male, n (%) | 197 (77.9%) | 244 (81.3%) | 0.312 |
| Age, median (IQR) | 55 (39, 66) | 55 (40.7, 65) | 0.919 |
| Family history of psoriasis, n (%) | 52 (20.6%) | 56 (18.7%) | 0.577 |
| BMI, median (IQR) | 26.3 (23.9, 29.3) | 26.3 (24.1, 29.1) | 0.781 |
| smoking, n (%) | 90 (35.6%) | 137 (45.7%) | 0.016 |
| Drinking, n (%) | 19 (7.5%) | 67 (22.3%) | < 0.001 |
| **Comorbidity** |  |  |  |
| Hypertension, n (%) | 96 (37.9%) | 123 (41%) | 0.464 |
| Type 2 diabetes mellitus, n (%) | 43 (17%) | 56 (18.7%) | 0.610 |
| PSA, n (%) | 59 (23.3%) | 67 (22.3%) | 0.783 |
| **Evaluation of psoriasis** |  |  |  |
| Duration (years), median (IQR) | 10 (6, 20) | 10 (6, 20) | 0.992 |
| BSA (%), median (IQR) | 30.0 (16.8, 42.0) | 30.0 (15.0, 40.0) | 0.502 |
| PASI, median (IQR) | 16.0 (12.0, 21.5) | 15.2 (11.5, 21.0) | 0.490 |
| **Hematological results** |  |  |  |
| Leukocyte (*10^9^/L), median (IQR) | 6.6 (5.7, 7.8) | 6.6 (5.6, 7.8) | 0.918 |
| Thrombocyte(*10^9^/L), median (IQR) | 210.0 (174.0, 264.0) | 212.0 (175.8, 262.3) | 0.876 |
| Lymphocyte(*10^9^/L), median (IQR) | 1.9 (1.5, 2.3) | 1.9 (1.5, 2.3) | 0.835 |
| Neutrophil(*10^9^/L), median (IQR) | 3.8 (3.3, 5.0) | 3.9 (3.3, 5.0) | 0.887 |
| NLR, median (IQR) | 2.0 (1.6, 2.8) | 2.033 (1.6, 2.8) | 0.931 |
| dNLR, median (IQR) | 1.5 (1.2, 1.9) | 1.4936 (1.2, 2.0) | 0.931 |
| SII, median (IQR) | 426.6 (316.7, 652.2) | 434.1 (317.4, 668.8) | 0.878 |
| Fasting blood glucose (mmol/L), median (IQR) | 5.3 (4.9, 6.1) | 5.4 (4.9, 6.4) | 0.164 |
| Triglyceride (mmol/L), median (IQR) | 1.6 (1.2, 2.2) | 1.7 (1.3, 2.3) | 0.132 |
| TyG, median (IQR) | 9.4 (8.9, 9.8) | 9.4 (9.1, 9.9) | 0.105 |
| TyG-BMI, median (IQR) | 249.1 (220.9, 276.1) | 250.6 (224.17, 276.2) | 0.413 |

NAFLD, non-alcoholic fatty liver disease; MAFLD, metabolic-associated fatty liver disease; IQR, interquartile range; BMI, body mass index; PSA, psoriatic arthritis; BSA, body surface area; PSAI, psoriasis area and severity index; NLR, neutrophil-to-lymphocyte ratio; dNLR, derived neutrophil-to-lymphocyte ratio; SII, Systemic immune inflammation Index; TyG, triglyceride-glucose; TyG-BMI, triglyceride glucose-body mass index.
